# Identifying potential drug targets for myocardial infarction through Mendelian randomization

**DOI:** 10.1371/journal.pone.0313770

**Published:** 2024-12-23

**Authors:** Xiangyou Yu, Shasha Liu

**Affiliations:** 1 Department of Endocrinology, Shaanxi Provincial People’s Hospital, Xi’an, P. R. China; 2 Department of Cardiology, Shaanxi Provincial People’s Hospital, Xi’an, P. R. China; Neyshabur University of Medical Sciences, ISLAMIC REPUBLIC OF IRAN

## Abstract

**Background:**

This study explored the associations between plasma and cerebrospinal fluid (CSF) proteins and myocardial infarction (MI) risk. Identifying specific proteins as biomarkers for MI could enhance our understanding of disease mechanisms and inform clinical practice.

**Methods:**

We combined protein quantitative trait loci (pQTL) data for plasma and CSF proteins with genome-wide association study (GWAS) summary statistics for MI. Mendelian Randomization (MR) analyses were conducted to establish causal relationships, supported by Bayesian colocalization and Spearman correlation analyses. For plasma proteins, we used pQTL data from Cheng et al. to select 738 cis-acting SNPs associated with 734 proteins. The "TwoSampleMR" method and inverse-variance weighted MR were applied for evaluations.

**Results:**

In plasma, CD8A and HDHD2 were identified as protective factors against MI, while DPEP1 was linked to increased risk. In CSF, CD30 Ligand was associated with MI risk. Bayesian colocalization supported the association for CD8A in plasma. No significant correlation was found between plasma and CSF results, suggesting distinct mechanisms for these biomarkers.

**Conclusion:**

Our study identified several plasma and CSF proteins linked to MI risk, offering new insights into the disease’s biological underpinnings. These findings could guide future research on MI biomarkers and contribute to improved prevention and treatment strategies.

## Introduction

Myocardial infarction (MI), commonly known as a heart attack, represents a major contributor to the global burden of cardiovascular disease. The prevalence of MI is estimated at 3.8% in individuals under 60 years of age and rises to 9.5% in those over 60 [1]. In the United States alone, nearly one million cases of MI are reported annually, leading to 300,000 to 400,000 deaths. Diagnosis typically includes assessing blood pressure, pulse, and body temperature, alongside diagnostic tests such as electrocardiograms (ECG/EKG) and blood tests [2]. Standard treatment for MI often involves antiplatelet agents, anticoagulants, nitrates, β-blockers, and statins. In some cases, percutaneous coronary intervention (PCI) is employed to reopen obstructed coronary arteries, while thrombolytic agents, such as tissue plasminogen activators, may be administered to dissolve existing clots [3].

Despite advancements in diagnosis and treatment, there are still limitations in the research and management of MI. Some challenges include: inappropriate disease models—preclinical MI research often employs animal models that may not accurately represent human conditions [4]; frequent alterations in surgical procedures—changing surgical techniques make it difficult to compare results across studies and to apply research findings to clinical practice; insufficient reporting transparency—poorly reported methods and results may hinder the reproducibility and generalizability of research findings [4]; and misdiagnosis—current regulatory methods for defining myocardial troponin clinical decision values are limited and may result in misdiagnosis of acute myocardial infarction (AMI), potentially harming patients. MI is a common and life-threatening condition with significant global impact [5]. While progress has been made in diagnosis and treatment, there remain limitations that need to be addressed in research and management to improve patient outcomes and alleviate the burden of cardiovascular diseases [6]. Mendelian randomization (MR) is a genetic epidemiology method that utilizes genetic variations as instrumental variables to investigate causal relationships between exposures and outcomes [7]. Genome-wide association studies (GWAS) are employed to identify genetic variations across the genome, such as single nucleotide polymorphisms (SNPs), that are associated with specific diseases or phenotypes [8]. When combined, MR and GWAS can effectively identify key disease targets, offering insights into the underlying mechanisms of disease and providing new strategies for prevention and treatment [9]. In this study, our goal is to identify proteins in plasma and cerebrospinal fluid (CSF) that could serve as potential therapeutic targets for myocardial infarction (MI). We begin by utilizing GWAS data from 11 plasma pQTLs identified in Zheng’s study, along with 6 plasma and CSF pQTLs from Yang’s research, to pinpoint MI-related proteins through MR analysis [10]. These findings are then validated using reverse causality tests, Bayesian co-localization, and phenome-wide association scans. Following this, we construct interaction networks between the identified proteins, linking those found in plasma and CSF, and comparing them with existing MI drug targets. Finally, we employ the FinnGen cohort’s GWAS data for external validation to reinforce our conclusions [11].

## Methods

### Quantitative trait loci of cerebrospinal fluid and plasma proteins

Data on CSF pQTLs was sourced from a research conducted by Yang and colleagues, which listed 274 pQTLs associated with 184 CSF proteins [12]. e incorporated only the pQTLs meeting the subsequent criteria: (i) outside of the major histocompatibility complex (MHC) zone (chr6, 26–34 Mb); (ii) showing a significant genome-wide link (P < 5 × 10^-8); (iii) having an independent relation [Linkage Disequilibrium (LD) grouping (r^2 < 0.001)]; (iv) functioning as cis-acting pQTLs [13]. As a result, 154 corresponding cis-pQTLs for 154 proteins were pinpointed. For initial explorations, plasma pQTL data was extracted from research by Cheng and team, which consolidated findings from five prior GWA [14]. Adhering to the selection guidelines mentioned for the CSF pQTL dataset, 738 cis-oriented SNPs associated with 734 proteins were selected [10]. All data were cross-referenced with original files to ensure reliability. For more detailed information on the selection of instrumental variables, please refer to S1 File Furthermore, relevant gene data were downloaded from the decode database for external validation. When selecting instrumental variables, we only included single nucleotide polymorphisms (SNPs) with significant effects and sufficient statistical power. To achieve this, we set a minimum F-statistic threshold of 10 to reduce the influence of weak instruments and thereby avoid bias caused by weak instrumental variables. To ensure the specificity of the instrumental variables, we applied methods such as MR-Egger and weighted median to test for horizontal pleiotropy. These methods help identify and adjust for instrumental variables that may influence myocardial infarction through multiple causal pathways. For instrumental variables that exhibited potential horizontal pleiotropy, we further excluded them from the analysis to ensure the reliability of the results.

### GWAS summary statistics for myocardial infarction

For the main analyses, summary statistics were derived from a GWAS dataset (ID: ukb-e-I21_CSA) involving 115,803 individuals of European descent (nCase = 8502, nControl = 8876) [15]. For external validation, summary statistics were obtained from FinnGen’s study (nCase = 21609, nControl = 285621, R8 version) [16].

### Mendelian randomization analysis

In our research, we treated plasma and CSF proteins as influencing factors and MI as the end result, leveraging the "TwoSampleMR" method for MR evaluations [17]. For proteins with a singular pQTL, Wald ratios were the go-to. However, when faced with multiple genetic tools, we adopted the inverse-variance weighted MR (MR-IVW) and subsequently conducted a heterogeneity assessment [18]. To determine the odds ratios (OR) linked to an elevated MI risk, we considered the standard deviation (SD) increment in plasma protein concentrations and a tenfold surge in CSF protein levels.

For our core assessments, we incorporated the Bonferroni adjustment to cater for multiple testings, setting a P-value boundary at 0.005 to earmark key results for deeper exploration. MR was executed solely on proteins shortlisted for external validation, marking the P-value limit at 0.05. In validating our initial outcomes, we employed consistent variant approaches from our primary studies and pivotal variant methods, harnessing SNPs with genome-wide significance as genetic determinants.

### Reverse causality tests

MI genetic instruments were selected from MI GWAS based on the same pQTL screening criteria to perform bidirectional MR analyses, aiming to identify potential reverse causality. Complete summary data for the proteins were obtained from three previous studies. The effects were estimated using MR-IVW, MR-Egger, weighted median, simple mode, and weighted mode methods. Steiger filtering was applied to confirm the directionality of the relationship between proteins and MI. Results were considered statistically significant at P < 0.05 [19].

### Bayesian colocalization analysis

We applied Bayesian colocalization analysis to assess the likelihood that two traits share common causal variants, using the "coloc" toolset with standard settings. As previously mentioned, this Bayesian approach provides posterior probabilities for five scenarios regarding the overlap of a single variant between two traits. In our analysis, particular emphasis was placed on hypothesis 4 (PPH4), which suggests that both the protein and MI are associated with the same genomic region through shared variants [20]. We applied both coloc.abf and coloc.susie methods. Genes showcasing a PPH4 exceeding 80% as determined by either method were earmarked as having supportive evidence for colocalization [21].

### Phenome scans

We also conducted phenome scans, searching previous GWAS to reveal associations of identified pQTLs with other traits. Phenome scans were conducted through "phenoscanner" and studies by Ferkingstad et al [22]. on GWAS plasma proteome. When SNPs fulfilled the following requirements, they were deemed pleiotropic: The findings indicate that: (i) the relationship was genome-wide significant (P < 5 x 10–8); (ii) the European population was the subject of the GWAS; and (iii) the SNP is linked to all known MI risk factors, such as metabolic characteristics, proteins, or clinical aspects. Furthermore, to identify possible relationships between the pQTLs for the selected proteins, LD r2 was computed [23].

### Comparative analyses and protein-protein interaction networks

We hypothesized that, due to the blood-brain barrier, there would be minimal correlation between plasma and CSF-identified pQTLs. To test this, we conducted Spearman correlation analyses on the effect estimates from CSF and plasma identified through MR analyses. Various P-value thresholds were applied to examine whether the correlation changed with increasing levels of significance [21].

Protein-protein interaction (PPI) networks were explored to identify associations with MI risk in CSF and plasma analyses (main MR analyses, P < 0.05). Our aim was to investigate how the prioritized proteins interact with each other and whether proteins identified from plasma data could interact with those found in CSF data [24]. Current drugs targeting identified potentially pathogenic proteins were also searched. All PPI analyses were conducted using the STRING database version 11.5, with a minimum required interaction score of 0.4. Moreover, MR was conducted through the Wald ratio method and Bayesian colocalization through coloc.abf, both with prioritized proteins as exposures and outcomes. MR with P < 0.05 was considered as potential interaction, and PPH4 > 0.8 as potential colocalization. Lastly, single-cell data were combined to analyze the distribution of key genes in single cells [25]. Annotation was performed on the single-cell dataset GSE180678, which includes a sample from a disease condition. The distribution of key genes across various cells was analyzed [26].

## Results

### Identifying pathogenic proteins for MI in the proteome

**Fig 1:**
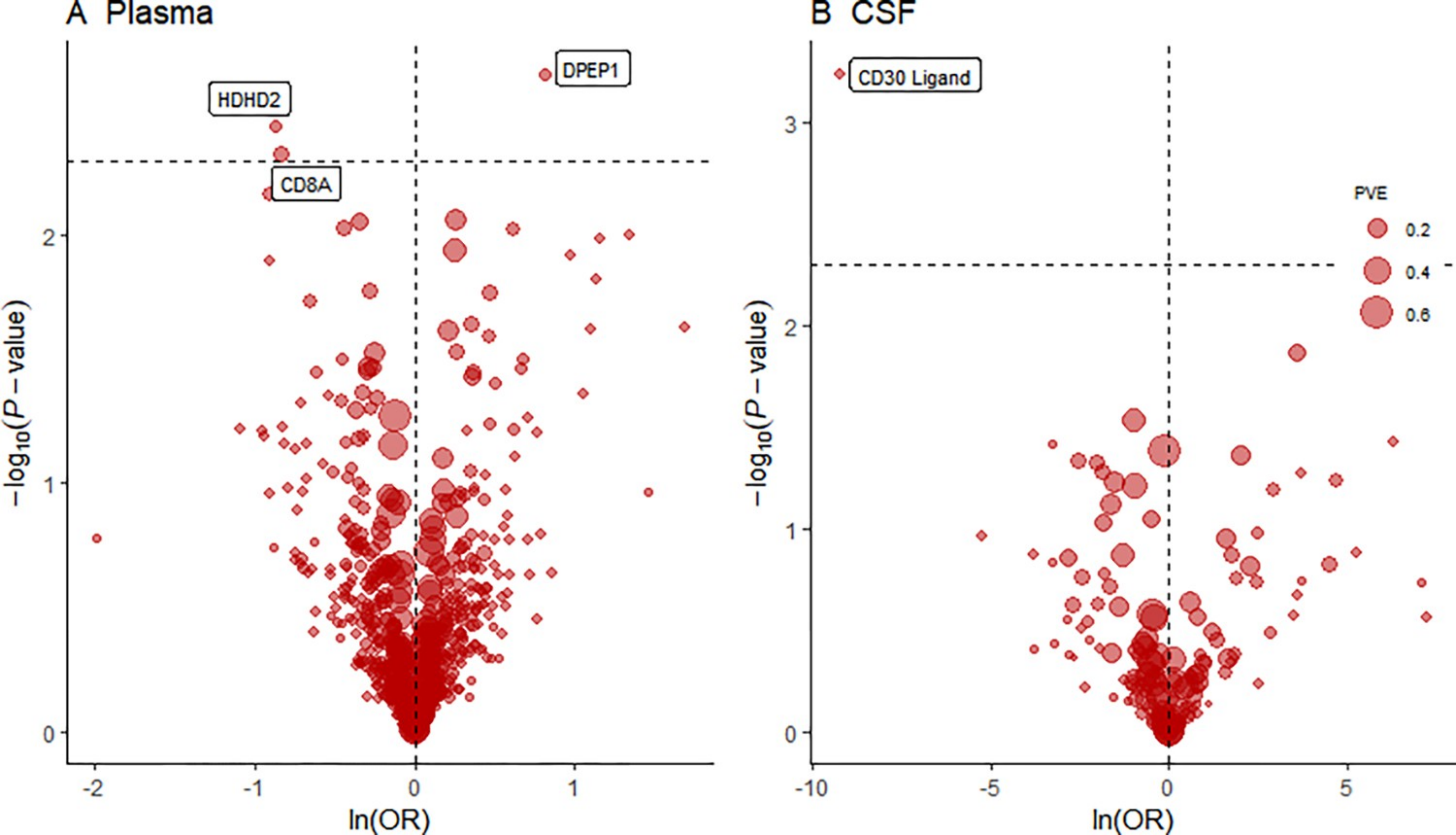
Volcano plots display the MR findings related to MI risk for 734 blood plasma proteins (A) and 154 cerebrospinal fluid (CSF) proteins (B). Both plots (A and B) present MR analysis of MI risk in relation to protein levels, utilizing either the Wald ratio or inverse-variance weighted approaches. The MI risk increase is shown per standard deviation rise in blood protein levels and every 10-fold elevation in CSF protein concentrations. A reference black dashed line indicates a P-value of 5.63×10^−5 (0.05 divided by 888). "ln" stands for natural logarithm, while "PVE" denotes the proportion of variance explained.

In our study, Mendelian Randomization (MR) analyses identified several proteins in plasma and cerebrospinal fluid (CSF) that may influence the risk of myocardial infarction (MI). In plasma, CD8A (UniProt ID: P01732, SNP: rs111976570, effect allele: A) was associated with a reduced MI risk, with an Odds Ratio (OR) of 0.43 (95% Confidence Interval [CI]: 0.24–0.77) and a P-value of 4.70E-03. In contrast, DPEP1 (UniProt ID: P16444, SNP: rs423135, effect allele: G) was linked to an increased MI risk, with an OR of 2.25 (95% CI: 1.34–3.78) and a P-value of 2.24E-03. HDHD2 (UniProt ID: Q9H0R4; V9HW73, SNP: rs75228657, effect allele: G) also demonstrated a protective trend against MI, with an OR of 0.42 (95% CI: 0.23–0.75) and a P-value of 3.63E-03. In CSF, CD30 Ligand (SNP: rs3181370, effect allele: G) showed significant association with MI risk, with a P-value of 5.88E-04 (Table 1 and Fig 1).

**Table 1:** A significant correlation between the MR data for plasma and cerebrospinal fluid proteins and MI.

Tissue	Protein	UniProt	SNP	allele	ci	P value	PVE	F statistics
Plasma	DPEP1	P16444	rs423135	G	2.24E-03	5.05%	170.09	Emilsson
Plasma	HDHD2	Q9H0R4; V9HW73	rs75228657	G	3.63E-03	4.80%	166.57	Sun
CSF	CD30 Ligand	P32971	rs3181370	G	5.88E-04	5.46%	48.22	Yang
Tissue	Protein	UniProt ID	SNP	allele	P value	PVE	F statistics	Author
Plasma	CD8A	P01732	rs111976570	A	4.70E-03	9.46%	334.54	Emilsson
Plasma	DPEP1	P16444	rs423135	G	2.24E-03	5.05%	170.09	Emilsson
Plasma	HDHD2	Q9H0R4; V9HW73	rs75228657	G	3.63E-03	4.80%	166.57	Sun

### Co-localization analysis for Myocardial Infarction (MI)

Following co-localization analysis for myocardial infarction (MI), we focused on four potentially relevant genes: DPEP1, CD8A, CD30 Ligand, and HDHD2. Based on Bayesian co-localization results, the posterior probability H4 for DPEP1 was 5.46%, suggesting a potential causal relationship with MI, but not strong enough to be considered compelling (PPH4 = 0.0546). CD8A showed a much stronger signal, with an H4 posterior probability of 35.5%, indicating a robust causal association with MI (PPH4 = 0.355). In contrast, CD30 Ligand had a very low H4 posterior probability of 2.13%, indicating a negligible causal relationship with MI (PPH4 = 0.0213). HDHD2 had a posterior probability of 5.27%, suggesting a modest degree of causal relationship, though the evidence is not definitive (PPH4 = 0.0527). No clear correlation was observed among these four genes. Overall, CD8A demonstrated the strongest causal link to MI, while CD30 Ligand had the weakest, with DPEP1 and HDHD2 presenting intermediate but inconclusive evidence. Further investigation is required to explore whether these genes are related to other diseases or physiological traits The specific information on colocalization can be found in Table 2.

**Table 2:** Presentation of co-localization analysis results.

Gene	PP.H0.abf	PP.H1.abf	PP.H2.abf	PP.H3.abf	PP.H4.abf	Shared variant (%)
DPEP1	0	0.599	0	0.347	0.0546	5.46%
CD8A	0	0.469	0	0.176	0.355	35.50%
CD30 Ligand	0.427	0.176	0.266	0.11	0.0213	2.13%
HDHD2	0.0427	0.595	0.0207	0.289	0.0527	5.27%
Gene	PP.H0.abf	PP.H1.abf	PP.H2.abf	PP.H3.abf	PP.H4.abf	Shared variant (%)

**Fig 2:**
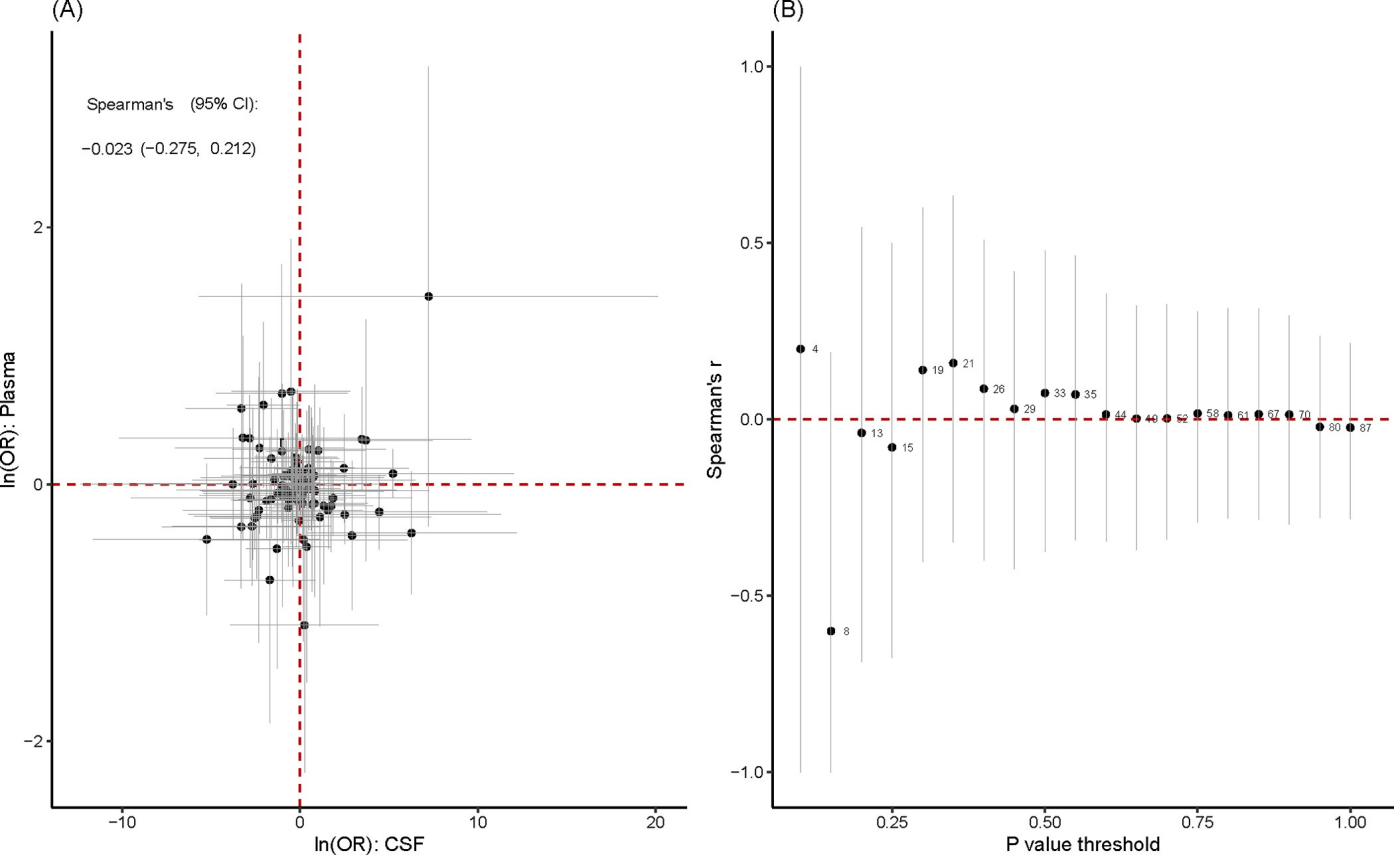
A on the basis of the 66 proteins present in both plasma and CSF, a correlation analysis was performed. The 95% confidence intervals for the primary MR analysis are shown by the grey horizontal and vertical lines. The result of the Spearman correlation is -0.023 (95% CI: -0.276, 0.212). B: The Spearman correlation values are computed because various P-value criteria are used to incorporate MR estimates. The number of common proteins under consideration is shown by the numbers next to the black dots.

The CSF and plasma MR data do not significantly correlate negatively at the protein level (Spearman correlation coefficient = -0.023, with no P-value cutoff). Furthermore, the negative connection continues but is not statistically significant when the number of proteins analyzed is limited by various P-value criteria (Fig 2).

### External validation of potential MI drug targets

**Fig 3:**
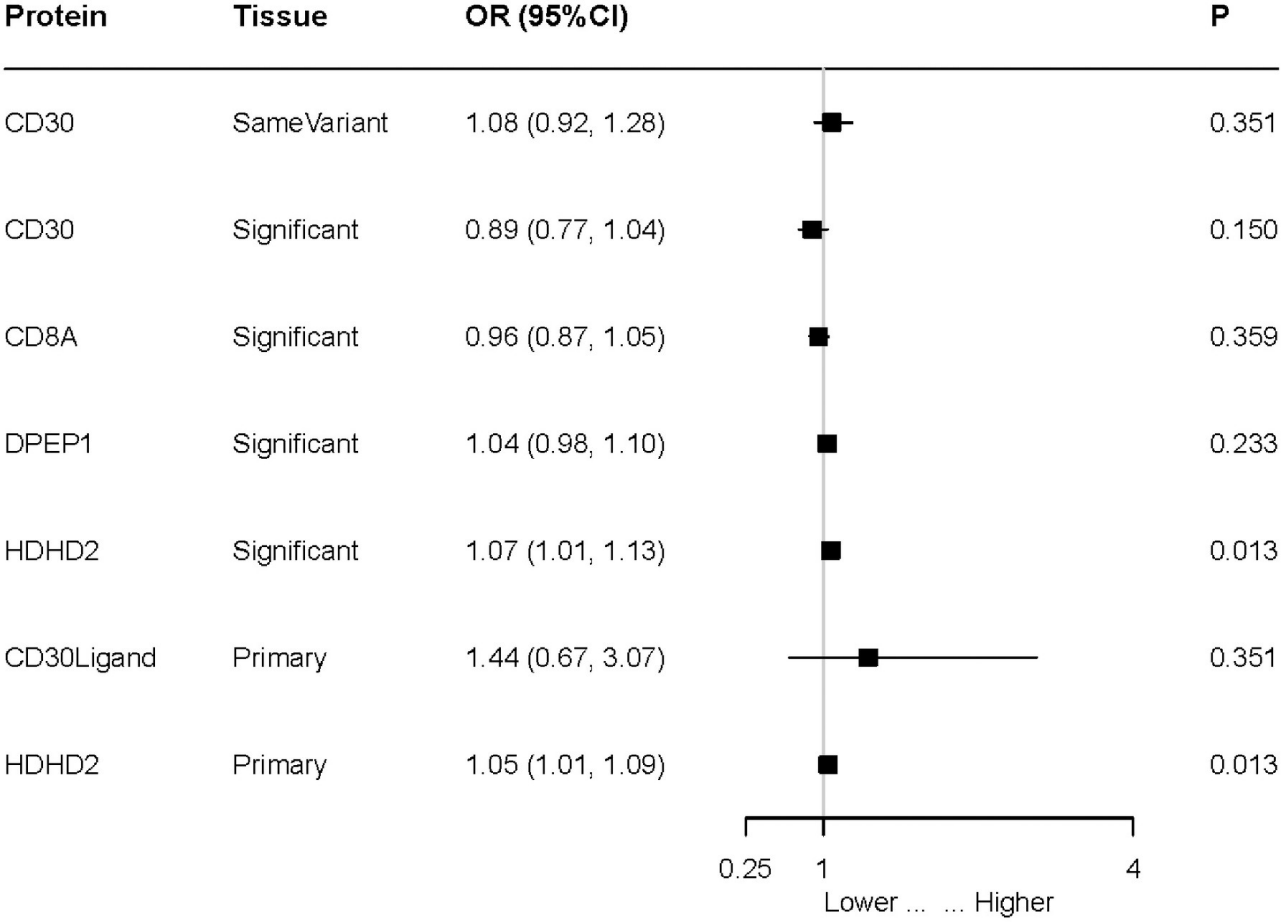
Establishing an external validation mechanism for the causal link between four putative causative proteins and MI using data from the FinnGen cohort. A Mendelian Randomization (MR) study is used to look at the possible cause-and-effect link between MI and these four proteins. The Odds Ratio (OR) per standard deviation (SD) increase in plasma protein levels and every 10-fold increase in CSF protein levels imply an elevated risk of MI.

While FCRL3 was only found to be associated with MI in the UK Biobank, MMEL1 showed a significant association with MI in the FinnGen cohort, using the same variant and significant variant strategies across different datasets to replicate the primary findings. HDHD2 was identified as having a significant impact. Under the significant variant strategy, an increase in HDHD2 was associated with an elevated risk of MI (OR = 1.07; 95% CI, 1.01–1.13; P = 0.013). In the primary dataset, the corresponding OR was 1.05 (95% CI, 1.01–1.09; P = 0.013). However, some external validation results were not significant. For example, the OR for CD30 under the same variant strategy was 1.08 (95% CI, 0.92–1.28; P = 0.351), and under the significant variant strategy, it was 0.89 (95% CI, 0.77–1.04; P = 0.150). The OR for CD8A under the significant variant strategy was 0.96 (95% CI, 0.87–1.05; P = 0.359) (Fig 3). The detailed results of the sensitivity analysis can be found in S2 File.

### Further analysis of key proteins

**Fig 4:**
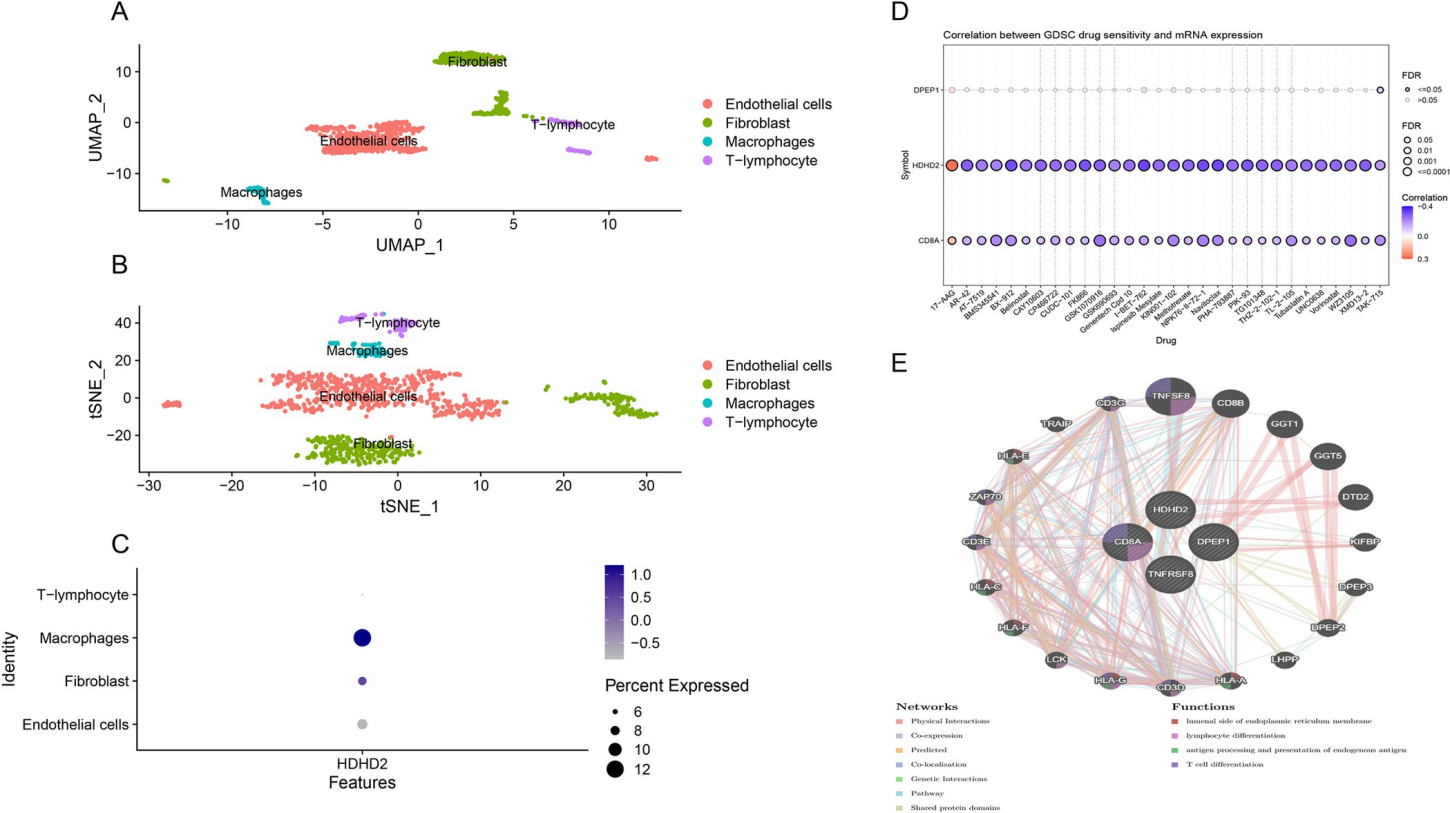
Further analysis of key proteins. Fig 4A and 4B show the uMAP and t-SNE annotation results from the dataset. Fig 4C illustrates the distribution of HDHD2, while Fig 4D presents drug sensitivity analysis. Finally, Fig 4E displays the interaction network of key proteins.

Through the annotation of single-cell data (GSE180678) for MI (Fig 4A and 4B), we observed a significant distribution of HDHD2 in macrophages, suggesting it may play a crucial role in the disease via these cells (Fig 4). The Protein-Protein Interaction (PPI) network revealed interactions between HDHD2 and related targets (Fig 4E). Additionally, we explored potential drug targets for these proteins using the GDSC database (Fig 4D).

## Discussion

In this study, we used Mendelian Randomization (MR) to identify several proteins in plasma and cerebrospinal fluid (CSF) that are potentially linked to myocardial infarction (MI) risk. Among the proteins identified, CD8A and HDHD2 were found to have protective effects against MI, while DPEP1 was associated with an increased risk of MI. We also used Bayesian co-localization analysis to investigate the causal relationships between these proteins and MI. Our results show that CD8A has the strongest causal association with MI, while CD30 Ligand has the weakest association. DPEP1 and HDHD2 showed intermediate yet inconclusive evidence of causality.

Additionally, single-cell RNA sequencing data revealed that HDHD2 is significantly distributed in macrophages, suggesting that it may play a crucial role in MI progression by modulating macrophage behavior. Further protein-protein interaction (PPI) analysis demonstrated that HDHD2 interacts closely with other key targets, indicating its potential as a therapeutic target. Single-cell analysis not only illuminated the cellular-level distribution of these proteins but also provided insights into the potential drug targets for MI treatment.

CD8A encodes the CD8α chain, which plays a critical role in immune defense and T-cell development [27]. Although there is limited research directly linking CD8A to MI, studies have shown that CD8+ T cells are involved in immune regulation following ischemic injury. CD8A may indirectly influence MI progression by regulating immune responses and interacting with cardiac macrophages during the heart repair process [28].

DPEP1, a protein associated with inflammation and poor cancer prognosis, has not been directly linked to MI, but its genetic variants are associated with cardiovascular disease risk markers [29]. DPEP1’s role in inflammation may influence MI indirectly through the regulation of systemic inflammatory responses. This study is the first to identify DPEP1 as a potential therapeutic target for MI, indicating its potential value in cardiovascular disease treatment [30].

The Role of HDHD2 in Myocardial Infarction: HDHD2 was identified as a novel therapeutic target in MI in our study. Although no previous studies have reported its role in MI, our findings suggest that HDHD2 may influence MI progression by regulating macrophage behavior [31]. Macrophages play a central role in the inflammatory response, tissue repair, and regeneration following MI. Recent advancements in single-cell RNA sequencing have revealed the heterogeneity of cardiac macrophages, showing that resident and recruited macrophages have distinct functions in the heart after MI. Targeting HDHD2 could help modulate macrophage activity, thereby enhancing the heart’s recovery after injury [32].

CD30 Ligand’s Role in MI: CD30 Ligand, a member of the tumor necrosis factor (TNF) superfamily, is primarily expressed in subsets of T and B cells and plays a role in immune regulation [33]. Although research on CD30 Ligand in cardiovascular disease is limited, its role in immune cell activation suggests it may be involved in the inflammatory response to MI. As a potential therapeutic target, CD30 Ligand may help modulate immune activity and reduce inflammation following MI [34].

Single-Cell Analysis Insights: The application of single-cell RNA sequencing allowed us to further investigate the complexity and diversity of macrophages following MI. It revealed that cardiac macrophages can be classified into resident and recruited subtypes, each with distinct roles in heart repair [35]. Resident macrophages are primarily involved in suppressing inflammation and promoting tissue repair, while recruited macrophages are more active in pro-inflammatory responses. HDHD2’s differential expression across these subtypes suggests that targeting it could provide a strategy to fine-tune macrophage behavior, thereby enhancing post-MI heart repair.

In summary, single-cell analysis provided new insights into HDHD2’s role in MI, highlighting its potential to regulate macrophage behavior and its importance in inflammation and tissue repair following cardiac injury. This opens up new avenues for future MI therapies, particularly in the development of macrophage-targeted treatments.

Our research has certain shortcomings. Initially, we examined the impacts of proteins from several research; however, discrepancies in measurement among these studies could have influenced the outcomes. The applicability of the analysis, including alternative MR algorithms, heterogeneity tests, and pleiotropy tests, is limited because all priority proteins have just one cis-acting SNP and no trans-pQTLs. The final results are not conclusive and require additional experimental verification, despite the fact that we did discover some connections between pathogenic proteins and therapeutic targets in already available multiple sclerosis drugs.

## Conclusion

In summary, our integrated analysis shows that the genetic determination levels of CD8A, DPEP1, HDHD2, and CD30 Ligand have a causal relationship with MI risk. The identified proteins may be attractive drug targets for MI, especially HDHD2. Further studies are needed to explore the roles of these candidate proteins in myocardial infarction.

## Supporting information

S1 File (XLSX)

S2 File (XLSX)
